# The profile of oxidative stress markers (arachidonic and linoleic acid derivatives) in patients with benign prostatic hyperplasia in relation to metabolic syndrome

**DOI:** 10.18632/aging.206187

**Published:** 2025-01-06

**Authors:** Weronika Ratajczak, Kinga Walczakiewicz, Maria Laszczyńska, Krzysztof Chmielowiec, Joanna Palma, Arleta Drozd, Anna Lubkowska, Olimpia Sipak

**Affiliations:** 1 Department of Functional Diagnostics and Physical Medicine, Pomeranian Medical University, Żołnierska, Szczecin 71-210, Poland; 2 Family Medicine Clinic, Bl. W. Kadłubka, Szczecin 71-521, Poland; 3 Department of Nursing, State University of Applied Sciences, Leśna, Koszalin 75-582, Poland; 4 Department of Hygiene and Epidemiology, Collegium Medicum, University of Zielona Góra, Zyty St., Zielona Góra 65-046, Poland; 5 Department of Biochemical Sciences, Pomeranian Medical University, Broniewskiego, Szczecin 71-460, Poland; 6 Department of Human Nutrition and Metabolomics, Pomeranian Medical University, Broniewskiego, Szczecin 71-460, Poland; 7 Department of Obstetrics and Pathology of Pregnancy, Pomeranian Medical University, Żołnierska, Szczecin 71-210, Poland

**Keywords:** benign prostatic hyperplasia (BPH), metabolic syndrome (MetS), lipid markers, inflammation, fatty acids derivatives

## Abstract

So far, it has been proven that benign prostatic hyperplasia (BPH) is strongly associated with inflammation resulting from, i.a. the presence of infectious agent, autoimmune disease, aging process and lipid disorders associated with metabolic syndrome (MetS). We analyzed the association between serum eicosanoides (HETE, HODE, lipoxins, prostaglandin, and leucotrien) in aging man with benign prostatic hyperplasia (BPH) and healthy controls. The study involved 219 men (with BPH, n = 144; healthy controls, n = 75). We assessed the content arachidonic and linoleic acid derivatives in the serum samples of the study participants using liquid chromatography (HPLC).

The levels of: RvE1 (p < 0.001); LXA_4_ 5S,6R,15R (p = 0.001); 10S,17R-DiDHA (p < 0.001); MaR1 (p = 0.002); 9S-HODE (p < 0.05); 15S-HETE (p < 0.05); 12S-HETE (p < 0.001); 5-oxoETE (p < 0.05) and 5-HETE (p < 0.001) were significantly higher in patients with BPH than in the control group. PGE2 (p = 0.007), LTB_4_ (p < 0.001), and 18RS-HEPE (p < 0.001) were significantly higher in control group.

We also analyzed the relationship between LXA_4_ 5S,6R,15R serum levels of oxidative stress markers and concomitance of MetS. We noticed a relationship between levels and MetS (F1216 = 6.114965, p = 0.01).

Our research results suggest that pro-inflammatory mediators and suppressors of inflammation are involved in the development of BPH, but their exact contribution has yet to be investigated.

## INTRODUCTION

Benign prostatic hyperplasia (BPH) is a prevalent disease among the aging male population worldwide. Clinical symptoms of BPH include lower urinary tract symptoms (LUTS), as well as prostate volume enlargement (BPE) and bladder outlet obstruction (BOO) [1–3]. The precise mechanism of BPH pathophysiology and development remains incompletely understood. Nevertheless, there are several risk factors that have been shown to increase the likelihood of developing BPH in men. Such factors include age, hyperinsulinemia and insulin resistance, obesity, steroid hormone disorders, and the influence of genetic factors. In recent years, there has been a notable increase in research on metabolic syndrome (MetS) - a factor increasing the risk of BPH and involved in its development. Abundant scientific evidence points to the role of metabolic disorders [4]. Furthermore, there is mounting evidence that links the onset of inflammation with the development of prostate diseases, including benign prostatic hyperplasia and prostate cancer [5]. BPH and MetS are important factors influencing the health quality of life (HRQoL) index in aging men [6, 7]. Metabolic syndrome (MetS) abnormalities lead to systemic inflammation and oxidative stress [8]. Typical features of MetS include disturbed lipid parameters: increased levels of low-density lipoproteins (LDL) and triglycerides (TG), as well as decreased levels of high-density lipoproteins (HDL). This dyslipidemia results in the production of interleukin-8 (IL-8) [9, 10]. Genetic and environmental factors that influence the pathogenesis of metabolic syndrome are also regarded as risk factors for chronic disease. Dietary fatty acids may be an important contributor to the evolution or prevention of MetS [11]. Previous studies of MetS patients have shown a link between serum fatty acids level and a variety of diseases, including nonalcoholic fatty liver disease (NAFLD) [12], chronic kidney disease [13], systemic lupus erythematosus (SLE) [14], and arteriosclerosis [15]. There are studies indicating the effect of fatty acid on hormonal and biochemical parameters in patients diagnosed with BPH. Preliminary evaluation and analysis of patients with BPH showed that metabolic syndrome may contribute to changes in the concentrations of polyunsaturated fatty acids (PUFA) [9].

Polyunsaturated fatty acids (PUFAs) can be classified according to the number of carbon atoms that follow the final double bond in the fatty acid chain. Docosahexaenoic acid (DHA) and eicosapentaenoic acid (EPA) (omega-3 (ω-3)) contain n-3 carbon atoms. Arachidonic acid (AA) and linoleic acid (LA) (omega-6 (ω-6)) contain n-6 carbon atoms [16].

**Figure 1:**
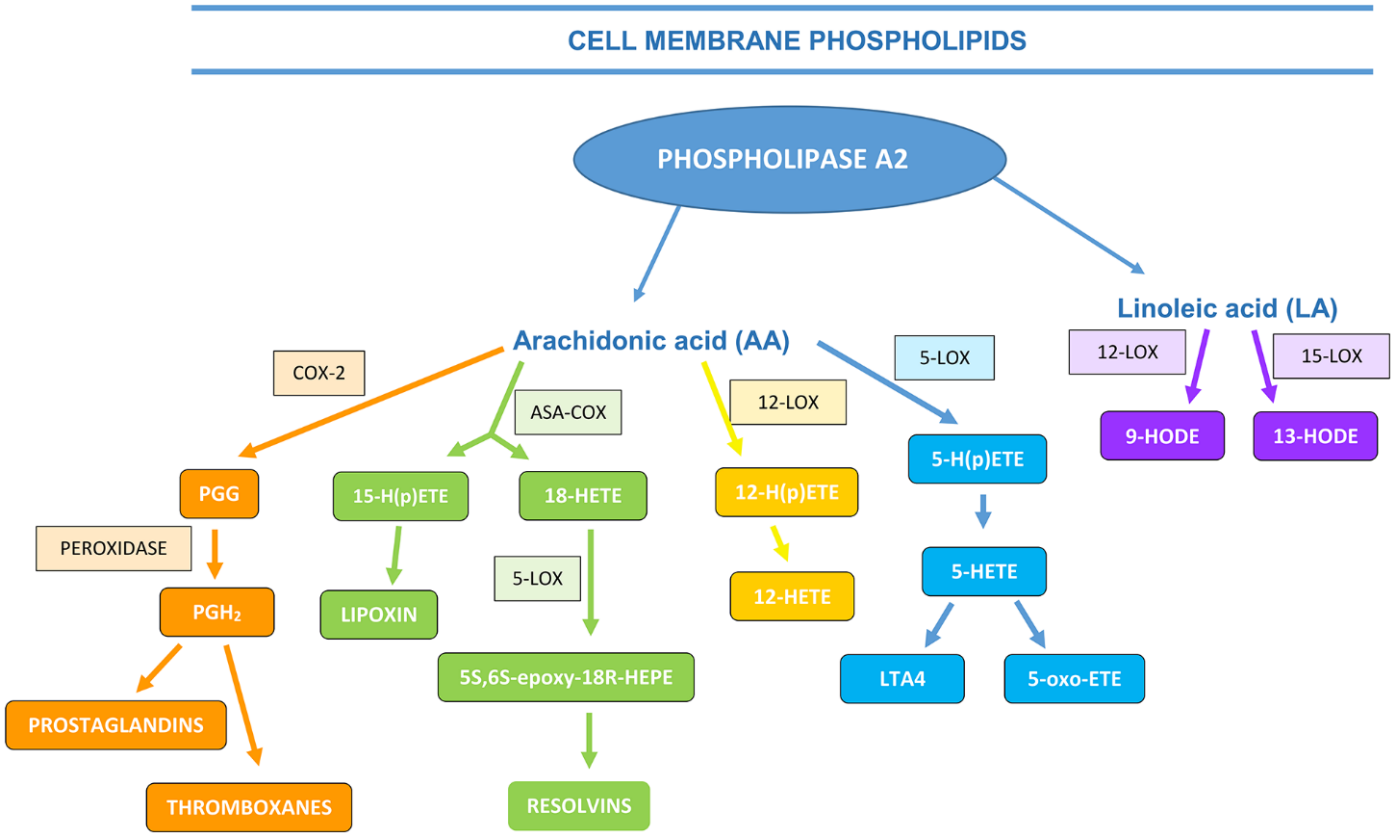
The formation of AA and LA metabolites (ASA-COX―acetylsalicylic acid (aspirin) cyclooxygenase, PGG―prostaglandin G, LOX―lipoxygenase, HETE―hydroxyeicosatetraenoic acids, HODE―hydroxyoctadecadienoic acid (based on Calder, 2010, 2013; the authors’ own modification)).

PUFA, AA, LA and its metabolites are important in regulating the course of inflammatory processes and diseases resulting from them. Three distinct enzyme systems may be involved in AA metabolism, namely cyclooxygenases (COX), lipoxygenases (LOX) and cytochrome (CYP) P450 enzymes, generating a broad spectrum of biologically active mediators derived from fatty acids. The oxidation products of arachidonic acid include: eicosanoids: prostanoides – prostaglandins (PGs), prostacyclin (PGI), thromboxane (TX); leukotrienes (LTs); lipoxines (LX) and hydroxyeicosatetraenoic acids (HETEs) [17]. Whereas, the products of LA oxidation are stable hydroxyoctadecadienoic acids (HODEs) [18] as shown in Figure 1.

Prostaglandin E2 (PGE2) is a member of the prostanoid lipid class, a subcategory of eicosanoids that are formed through the oxidation of essential fatty acids (EFA) with a 20-carbon chain. Prostaglandin E2 (PGE2) is a lipid that plays a pivotal role in the inflammatory response, including the progression of neoplastic processes. PGE2 exerts effects on cell proliferation and the process of apoptosis, in addition to influencing angiogenesis and immune responses. Additionally, PGE2 has been demonstrated to promote pyrogenic effects and has strong immunosuppressive properties, which contribute to the resolution phase of acute inflammation. This, in turn, facilitates tissue healing and the establishment of homeostasis [19].

Leukotrienes, 5-HETE and 5-oxo-ETE are regarded as potent mediators of inflammation. They are pivotal intermediates in the 5-LOX pathway, which induces macrophage infiltration, thereby instigating an inflammatory response [20]. The activating effect of 5-HETE on human neutrophils is approximately 100 times weaker than that of 5-oxo-ETE. Furthermore, 5-oxo-ETE has been demonstrated to facilitate neutrophil aggregation and act as a chemoattractant for human eosinophils and basophils. Additionally, 5-oxo-ETE has been shown to play a role in the induction of monocyte migration. It has also been confirmed that 5-oxo-ETE is involved in the pathogenesis of various diseases, including asthma, allergy, and cancer development [20].

Lipoxins (LX) are lipid-derived anti-inflammatory mediators. LX exert anti-inflammatory effects, including the inhibition of pro-inflammatory factor expression while simultaneously promoting the expression of anti-inflammatory factors. Additionally, LX has been demonstrated to inhibit the inflammatory action of neutrophils and promote their phagocytosis by macrophages [21]. LXs help to dampen inflammation and promote tissue repair. Their role is confirmed in chronic inflammation, cancer, respiratory tract, neurodegenerative and liver diseases, endometriosis, and retinal degeneration [22].

The metabolite 15-HETE of arachidonic acid (AA) is frequently elevated during inflammatory conditions. 12-HETE is an arachidonic acid metabolite of 12-LOX, and one of its biological functions is to act as a chemoattractant for neutrophils. [23]. This acid also affects the stimulation and inhibition of platelets [24]. 12(S)-HETE is also involved in the motor phase of metastasis, acting via the GPR31 receptor. This phase consists of cell adhesion, spreading, secretion of proteases and regression of endothelial cells. Moreover, it also has a protective function against apoptosis and can stimulate angiogenesis. [25]. It has been demonstrated that 15S-HETE plays a role in regulating the function of vascular smooth muscle and endothelial cells. Furthermore, it may also be involved in cellular proliferation and the course of inflammation. In contrast to 12S-HETE, 15S-HETE may participate in protective and immune responses against cancer cells. In the context of prostate cancer, 15S-HETE may play a role in the inhibition of cell cycle progression in prostate cancer cells [24]. 16-R/S HETE is cytochrome P450-derived metabolite of AA metabolism. It is isolated from human PMNs (polymorphonuclear cells) and act as endogenous regulator for these cells [26]. Moreover, 16-HETE functions as neutrophil suppressor [27]. Given its vasodilatory properties, 16-HETE can be regarded as a potential therapeutic target in the management of cardiovascular disorders [26]. Vascular endothelial cells that are hypoxic are capable of producing 18R/S-HEPE. Moreover, it has been demonstrated that 18-HEPE possesses anti-inflammatory and anti-apoptotic properties [28]. 18-HEPE is cardioprotective by inhibition of macrophage-mediated proinflammatory activation of cardiac fibroblasts [29, 30]. Moreover, there is a report that 18-HEPE is a potential anticancer metabolite [31]. Another property of 18-HEPE is the increase in BDNF mRNA expression in Müller glia cells, which affects the ameliorate retinal neuronal cell dysfunction [32].

Hydroxyoctadecadienoic acids (9-HODE and 13-HODE) play a pivotal role in cell signaling and the regulation of inflammatory processes, which occurs through the involvement of adhesion molecules. Additionally, these acids are associated with the degranulation and chemotaxis of neutrophils. Moreover, during the course of processes involving HODE acids, superoxide is produced by macrophages and receptor PPAR-γ (gamma) is activated. Moreover they are involved in inhibition of protein kinase C. The role of these acids has been confirmed in the regulation of inflammation occurring in the course of metabolic syndrome, atherosclerosis, but also in the neoplastic process [33].

Maresins are effective anti-inflammatory mediators, which have a strong protective effect in inflammation, oxidative stress and immune diseases. They act as inhibitors of neutrophil infiltration and naive T cell differentiation. Furthermore, they inhibit the expression of proinflammatory cytokines, including interleukins 1β and 6, as well as TNF-α. Maresin 1 is derived from DHA20, and it is produced in macrophages, platelets and neutrophils [34].

Resolvins are synthesised from DHA, n-3DPA (docosapentaenoic acid), and EPA. They possess immunomodulatory functions, including the inhibition of leukocyte infiltration and the capacity to enhance phagocytosis by macrophages. Moreover, they are involved in the regulation of cytokine secretion, which mediates the reduction of inflammatory pain. Resolvins play a pivotal role in modulating the progression of inflammatory processes within the nervous system, joints, and blood vessels [35]. Resolvin E1 (RvE1) has been demonstrated to be involved in the mitigation of intestinal inflammation through the enhanced production of the anti-inflammatory cytokine IL-10 by macrophages [36].

Among the functions of RvE1, its action on reducing the level of leukocyte adhesion proteins (CD11 and CD18) and the activation of platelets are particularly noteworthy. Additionally, RvE1 induces the apoptosis of PMN cells, thereby limiting the extent of inflammation [29].

Resolvin D1 (RvD1), synthesised from docosahexaenoic acid (DHA), has been demonstrated to prevent organ damage occurring during inflammation of the lungs, kidneys, pancreas, liver and peritoneum (confirmed in an animal model). The anti-inflammatory effect of RvD1 may be mediated by inhibiting neutrophil recruitment and inflammatory cytokine production [37].

Proteins constitute a family of pro-resolving mediators. They are involved in combating bacterial and viral infections by increasing the efficacy of the killing process and, as a result, microbial clearance. Additionally, PD1 limits leukocyte infiltration, but also reduces NF-KB signaling, pro-inflammatory chemokines and cytokines, inflammasome formation and COX-2 activation. Furthermore, PD1 has been demonstrated to exert a robust neuroprotective effect. PD1 has been shown to facilitate the enhanced expression of antiviral interferon (lambda) in bronchial epithelial cells, which in humans may exert a considerable impact on the attenuation of inflammatory processes within lung tissue. [38].

This study represents one of the first to examine the levels of linoleic acid derivatives and anti- and pro-inflammatory mediators in patients with BPH and aging men without BPH.

The primary objective was to examine the profile of lipid markers of oxidative stress (arachidonic and linoleic acid derivatives) in the serum of patients with BPH and concomitant metabolic syndrome.

## RESULTS

Anthropometric measurements were performed in patients from both groups. No statistically significant differences were observed in body weight, BMI and waist circumference between patients from the control and BPH group (Table 1). Analysis of variance (ANOVA) was conducted on the control and study groups with respect to MetS, the results of which are presented in Table 2.

**Table 1:** Age, body measurements, lipid parameters and glucose in control and BPH group.

**Parameter**	**Control group (n = 75)**					**BPH group (n = 144)**					
	**Mean**	**Me**	**Min**	**Max**	**SD**	**Mean**	**Me**	**Min**	**Max**	**SD**	**p-value**
**Age [years]**	54.662	54.000	45.000	72.000	6.523	66.326	67.00	48.000	75.000	6.479	< 0.001*
**Body weight [kg]**	88.853	86.350	65.500	132.500	11.861	85.909	85.00	55.000	118.000	13.361	0.101
**BMI [kg/m^2^]**	28.479	27.643	21.634	38.714	3.789	28.198	28.038	18.591	42.607	3.836	0.794
**WC [cm]**	100.956	100.000	85.000	134.000	9.809	101.802	102.000	72.000	136.000	11.840	0.587
**Cholesterol [mg/dl]**	215.133	208.391	136.296	320.279	39.309	204.924	199.631	143.494	404.095	37.624	**0.059**
**TG [mg/dl]**	163.074	156.800	110.485	306.666	40.371	186.101	162.534	114.049	815.427	95.894	< 0.05*
**HDL [mg/dl]**	44.760	43.981	30.324	76.620	10.638	51.519	50.224	39.237	72.870	7.207	< 0.001*
**LDL [mg/dl]**	137.758	133.172	56.316	232.632	40.476	117.562	112.567	41.041	252.728	33.163	< 0.001*
**Glucose [mg/dl]**	100.654	99.416	85.430	161.125	10.504	82.064	82.811	49.424	155.686	15.237	< 0.001*

Me, median; Min, minimum; Max, maximum; SD, standard deviation; BPH, benign prostatic hyperplasia; n, number; BMI, body mass index; WC, waist circumference; TG, triglycerides; HDL, high density lipoprotein; LDL, low density lipoprotein; p, statistical significance; * –statistically significant parameter.

**Table 2:** The results of multivariate ANOVA of anthropometric parameters with regard to MetS in both groups (with and without BPH).

**Variables**	**BPH group**		**Control group**		**Multivariate ANOVA**			
	***without MetS*** ***(n = 75)***	***with MetS*** ***(n = 69)***	***without MetS*** ***(n = 40)***	***with MetS*** ***(n = 40)***	***Factor***	***F (value p)***	***η2***	***Power (alpha = 0.05)***
**Anthropometric parametres**								
**Age [years]**	X = 65.93 SD = 7.42	X = 66.75 SD = 5.28	X = 54.47 SD = 6.51	X = 54.85 SD = 6.61	**intercept**	**F_1220_ = 17729.30 (p < 0.001)**	**0.988**	**1.000**
					**BPH/control**	**F_1220_ = 165.21 (p < 0.001)**	**0.429**	**1.000**
					**MetS**	F_1220_ = 0.43 (p = 0.511)	0.002	0.100
					BPH/control*MetS	F_1220_ = 0.06 (p = 0.806)	0.001	0.057
**Body weight [kg]**	X = 80.26 SD = 11.41	X = 92.02 SD = 12.68	X = 83.94 SD = 9.10	X = 93.76 SD = 12.35	**intercept**	**F_1220_ = 11634.07 (p < 0.001)**	**0.981**	**1.000**
					**BPH/control**	F_1220_ = 2.77 (p = 0.097)	0.012	0.381
					**MetS**	**F_1220_ = 44.13 (p < 0.001)**	**0.167**	**0.999**
					BPH/control*MetS	F_1220_ = 0.35 (p = 0.556)	0.002	0.090
**BMI [kg/m^2^]**	X = 26.55 SD = 3.18	X = 29.89 SD = 3.69	X = 26.6 SD = 2.83	X = 30.34 SD = 3.72	**intercept**	**F_1220_ = 14365.20 (p < 0.001)**	**0.985**	**1.000**
					**BPH/control**	F_1220_ = 0.20 (p = 0.659)	0.001	0.073
					**MetS**	**F_1220_ = 57.44 (p < 0.001)**	**0.207**	**1.000**
					BPH/control*MetS	F_1220_ = 0.10 (p = 0.752)	0.001	0.061
**WC [cm]**	X = 95.52 SD = 10.87	X = 108.63 SD = 8.67	X = 95.75 SD = 7.25	X = 106.13 SD = 9.33	**intercept**	**F_1220_ = 24116.78 (p < 0.001)**	**0.990**	**1.000**
					**BPH/control**	F_1220_ = 0.73 (p = 0.392)	0.003	0.136
					**MetS**	**F_1220_ = 80.59 (p < 0.001)**	**0.268**	**1.000**
					BPH/control*MetS	F_1220_ = 1.10 (p = 0.294)	0.005	0.182

Lipid parameters in serum were measured in the control and study group (Table 1). Statistical analysis revealed that total cholesterol (p = 0.059), LDL cholesterol (p < 0.001) and glucose (p < 0.001) levels were statistically significantly higher in the healthy patients group. Conversely, the patients with BPH exhibited statistically higher TG (p < 0.05) and HDL cholesterol (p < 0.001) levels.

### Eicosanoids profile in BPH patients

A comparative analysis of serum levels of inflammatory markers in men with BPH (study group) and without BPH (control group) was performed. BPH patients were found to have statistically significantly higher levels of: *Rv*E1 (p < 0.001); PGE2 (p = 0.007), 10S,17R-DiDHA (p < 0.001); *MaR*1 (p = 0.002); 15S-HETE (p < 0.05); 12S-HETE (p < 0.001); 9S-HODE (p < 0.05); 5-oxo-ETE (p < 0.05) and 5-HETE (p < 0.001). At the same time, they had lower levels of LXA_4_ 5S,6R (p < 0.001), LTB4 (p < 0.001), and 18RS-HEPE (p < 0.001) (Table 3). In summary, in patients with BPH, pro-inflammatory factors statistically significantly predominated in the assessed eicosanoid profile.

**Table 3:** The comparison of eicosanoid levels between healthy volunteers and patients with BPH.

**Eicosanoids [μg/ml]**	**Control group (n = 75)**					**BPH patients (n = 144)**					**p-value**
	**Mean**	**Me**	**Min**	**Max**	**SD**	**Mean**	**Me**	**Min**	**Max**	**SD**	
**RvD1**	0.001	0.000	0.000	0.010	0.001	0.003	0.000	0.000	0.152	0.015	0.082
**RvE1**	0.135	0.107	0.023	0.813	0.122	0.275	0.206	0.00	7.351	0.612	< 0.001*
**PGE2**	7.585	4.799	0.756	79.464	10.347	10.561	7.280	0.000	79.112	10.616	0.007*
**LXA_4_ 5S,6R**	4.265	2.804	0.000	20.157	4.480	2.272	1.446	0.00	25.091	2.944	<0.001*
**LXA_4_ 5S,6R,15R**	0.006	0.000	0.000	0.169	0.028	0.002	0.000	0.00	0.090	0.012	0.081
**10S,17R - DiDHA (Protectin D1)**	0.112	0.085	0.000	0.554	0.095	0.184	0.153	0.00	1.794	0.195	< 0.001*
**LTB4**	0.087	0.075	0.000	0.265	0.050	0.045	0.036	0.00	0.901	0.076	< 0.001*
***MaR*1**	0.023	0.000	0.000	0.448	0.060	0.027	0.025	0.00	0.145	0.028	0.002*
**18 R/S-HEPE**	0.148	0.131	0.000	0.454	0.077	0.126	0.096	0.00	2.400	0.205	< 0.001*
**16 R/S-HETE**	0.388	0.205	0.000	2.711	0.548	0.380	0.297	0.00	2.936	0.521	0.921
**15 S-HETE**	0.581	0.543	0.222	1.561	0.214	0.933	0.624	0.00	7.651	1.017	< 0.05*
**12 S-HETE**	6.007	4.741	1.122	23.283	4.805	11.346	8.854	0.00	50.660	9.337	< 0.001*
**13 S-HODE**	0.081	0.076	0.018	0.206	0.044	0.109	0.076	0.00	1.986	0.172	0.227
**9 S-HODE**	0.080	0.076	0.019	0.249	0.047	0.107	0.087	0.00	0.939	0.097	< 0.05*
**5-oxo-ETE**	0.247	0.239	0.000	0.742	0.223	0.508	0.280	0.00	9.434	0.944	< 0.05*
**5-HETE**	0.092	0.087	0.043	0.175	0.028	0.212	0.123	0.00	4.528	0.403	< 0.001*

Me, median; Min, minimum; Max, maximum; SD, standard deviation; BPH, benign prostatic hyperplasia; n, number; RvE1, resolvin E1; PGE2, prostaglandin E2; RvD1, resolvin D1; LX, lipoxin; DiDHA, dihydroxydocosahexaenoic acid; LTB4, leukotriene B4; MaR1, maresin 1; HEPE, hydroxyeicosapentaenoic acid; HETE, hydroxyeicosatetraenoic acid; HODE, hydroxyoctadecadienoic acid; ETE, eicosatetraenoic acid.

### Metabolic syndrome is associated with levels of LXA_4_ 5S,6R,15R

Based on the results, multivariate analysis of variance (MANOVA) was carried out. A comparison of the groups (BPH patients vs. controls) in terms of LXA_4_ 5S,6R,15R levels and MetS (F_1216_ = 6.114965, p = 0.01), which accounted for 2.8% of the variance, showed significant results for the interaction. In the control group, MetS was associated with a decrease in serum leukotriene (LT) levels, unlike in the study group, whose members with MetS had higher serum LXA_4_ 5S,6R,15R levels than those without MetS. No impact of MetS on the concentrations of other lipid inflammatory markers was noted. The results of post-hoc analysis are shown in Tables 4, 5. Research results indicate that in patients with BPH, the elevated levels of an eicosanoid that suppresses the inflammatory response (LXA_4_ 5S,6R,15R) are associated with the occurrence of metabolic syndrome.

**Table 4:** The results of multivariate ANOVA of eicosanoid levels with regard to MetS in both groups (with and without BPH).

**LIPID MARKERS OF OXIDATIVE STRESS**	**BPH group**		**Control group**		**Multivariate ANOVA**			
	***without MetS (n = 75)***	***with MetS (n = 69)***	***without MetS (n = 40)***	***with MetS (n = 40)***	***Factor***	***F (value p)***	***η2***	***Power (alpha = 0.05)***
**RvD1 [μg/ml]**	X = 0.002 SD = 0.005	X = 0.006 SD = 0.022	X = 0.005 SD = 0.002	X = 0.000 SD = 0.000	**intercept**	**F_1215_ = 5.662540 (p < 0.001)**	**0.026**	**0.659**
					**BPH/control**	**F_1215_ = 4.499296 (p = 0.035)**	**0.021**	**0.560**
					**MetS**	F_1215_ = 0.842518 (p = 0.360)	0.004	0.150
					BPH/control*MetS	F_1215_ = 1.383782 (p = 0.241)	0.006	0.216
**RvE1 [μg/ml]**	X = 0.317 SD = 0.837	X = 0.229 SD = 0.147	X = 0.132 SD = 0.099	X = 0.139 SD = 0.144	**intercept**	**F_1216_ = 32.91312 (p < 0.001)**	**0.132**	**0.999**
					**BPH/control**	F_1216_ = 3.72673 (p = 0.055)	0.017	0.485
					**MetS**	F_1216_ = 0.32755 (p = 0.568)	0.002	0.088
					BPH/control*MetS	F_1216_ = 0.44148 (p = 0.507)	0.002	0.101
**PGE2 [μg/ml]**	X = 10.740 SD = 11.798	X = 10.364 SD = 9.266	X = 6.500 SD = 4.102	X = 8.728 SD = 14.236	**intercept**	**F_1215_ = 147.0181 (p < 0.001)**	**0.406**	**1.000**
					**BPH/control**	**F_1215_ = 3.8488 (p = 0.051)**	**0.018**	**0.497**
					**MetS**	F_1215_ = 0.3805 (p = 0.538)	0.002	0.094
					BPH/control*MetS	F_1215_ = 0.7574 (p = 0.385)	0.004	0.139
**LXA_4_ 5S,6R [μg/ml]**	X = 1.820 SD = 3.039	X = 2.765 SD = 2.775	X = 3.882 SD = 3.893	X = 4.668 SD = 5.049	**intercept**	**F_1216_ = 171.4006 (p < 0.001)**	**0.442**	**1.000**
					**BPH/control**	**F_1216_ = 15.6270 (p < 0.001)**	**0.067**	**0.976**
					**MetS**	**F_1216_ = 2.9773 (p = 0.09)**	**0.014**	**0.404**
					BPH/control*MetS	F_1216_ = 0.0250 (p = 0.874)	0.001	0.053
**LXA_4_ 5S,6R,15R [μg/ml]**	X = 0.001 SD = 0.009	X = 0.003 SD = 0.014	X = 0.012 SD = 0.038	X = 0.000 SD = 0.000	**intercept**	**F_1216_ = 9.117860 (p = 0.003)**	**0.040**	**0.852**
					**BPH/control**	F_1216_ = 2.041960 (p = 0.154)	0.009	0.296
					**MetS**	**F_1216_ = 3.903412 (p < 0.05)**	**0.018**	**0.503**
					BPH/control*MetS	**F_1216_ = 6.114965 (p = 0.01)**	**0.028**	**0.692**
**10S,17R - DiDHA (Protectin D1) [μg/ml]**	X = 0.184 SD = 0.224	X = 0.183 SD = 0.160	X = 0.092 SD = 0.066	X = 0.132 SD = 0.116	**intercept**	**F_1216_ = 154.1586 (p < 0.001)**	**0.416**	**1.000**
					**BPH/control**	**F_1216_ = 8.9985 (p = 0.003)**	**0.040**	**0.848**
					**MetS**	F_1216_ = 0.6305 (p = 0.428)	0.003	0.124
					BPH/control*MetS	F_1216_ = 0.7376 (p = 0.391)	0.003	0.137
**LTB4 [μg/ml]**	X = 0.051 SD = 0.102	X = 0.039 SD = 0.028	X = 1.820 SD = 3.039	X = 1.820 SD = 3.039	**intercept**	**F_1216_ = 185.5294 (p < 0.001)**	**0.462**	**1.000**
					**BPH/control**	**F_1216_ = 18.5361 (p < 0.001)**	**0.079**	**0.989**
					**MetS**	F_1216_ = 2.0341 (p = 0.155)	0.009	0.295
					BPH/control*MetS	F_1216_ = 0.0196 (p = 0.889)	0.001	0.052
***MaR*1 [μg/ml]**	X = 0.024 SD = 0.020	X = 0.031 SD = 0.033	X = 0.025 SD = 0.044	X = 0.022 SD = 0.074	**intercept**	**F_1216_ = 72.66671 (p < 0.001)**	**0.252**	**1.000**
					**BPH/control**	F_1216_ = 0.44679 (p = 0.505)	0.002	0.102
					**MetS**	F_1216_ = 0.15339 (p = 0.696)	0.001	0.068
					BPH/control*MetS	F_1216_ = 0.76298 (p = 0.383)	0.004	0.140

**Table 5:** The results of multivariate ANOVA of eicosanoid levels with regard to MetS in both groups (with and without BPH).

**LIPID MARKERS OF OXIDATIVE STRESS**	**BPH group**		**Control group**		**Multivariate ANOVA**			
	***without MetS*** ***(n = 75)***	***with MetS*** ***(n = 69)***	***without MetS*** ***(n = 40)***	***with MetS*** ***(n = 40)***	***Factor***	***F (value p)***	***η2***	***Power (alpha = 0.05)***
**18(RS)-HEPE [μg/ml]**	X = 0.150 SD = 0.276	X = 0.100 SD = 0.065	X = 0.160 SD = 0.082	X = 0.134 SD = 0.070	**intercept**	**F_1216_ = 124.8228 (p < 0.001)**	**0.366**	**1.000**
					**BPH/control**	F_1216_ = 0.8448 (p = 0.360)	0.004	0.150
					**MetS**	F_1216_ = 2.4625 (p = 0.118)	0.011	0.346
					BPH/control*MetS	F_1216_ = 0.2423 (p = 0.623)	0.0001	0.078
**16(RS)-HETE [μg/ml]**	X = 0.366 SD = 0.499	X = 0.396 SD = 0.546	X = 0.479 SD = 0.058	X = 0.293 SD = 0.509	**intercept**	**F_1216_ = 104.1667 (p < 0.001)**	**0.325**	**1.000**
					**BPH/control**	F_1216_ = 0.0043 (p = 0.948)	0.00002	0.050
					**MetS**	F_1216_ = 1.0657 (p = 0.303)	0.005	0.177
					BPH/control*MetS	F_1216_ = 2.0845 (p = 0.150)	0.010	0.301
**15(S)-HETE [μg/ml]**	X = 0.988 SD = 1.261	X = 0.872 SD = 0.661	X = 0.234 SD = 0.603	X = 0.559 SD = 0.190	**intercept**	**F_1216_ = 162.4929 (p < 0.001)**	**0.429**	**1.000**
					**BPH/control**	**F_1216_ = 8.6798 (p = 0.004)**	**0.039**	**0.835**
					**MetS**	F_1216_ = 0.4566 (p = 0.500)	0.002	0.103
					BPH/control*MetS	F_1216_ = 0.0905	0.0004	0.060
**12(S)-HETE [μg/ml]**	X = 11.664 SD = 9.724	X = 11.001 SD = 8.956	X = 6.613 SD = 5.250	X = 5.369 SD = 4.256	**intercept**	**F_1216_ = 227.5942 (p < 0.001)**	**0.513**	**1.000**
					**BPH/control**	**F_1216_ = 21.6362 (p < 0.001)**	**0.091**	**0.996**
					**MetS**	F_1216_ = 0.6888 (p = 0.407)	0.003	0.131
					BPH/control*MetS	F_1216_ = 0.0639 (p = 0.801)	0.0003	0.057
**13(S)-HODE [μg/ml]**	X = 0.116 SD = 0.230	X = 0.101 SD = 0.069	X = 0.083 SD = 0.045	X = 0.078 SD = 0.042	**intercept**	**F_1216_ = 87.96296 (p < 0.001)**	**0.289**	**1.000**
					**BPH/control**	F_1216_ = 1.91461 (p = 0.168)	0.009	0.281
					**MetS**	F_1216_ = 0.24612 (p = 0.620)	0.001	0.078
					BPH/control*MetS	F_1216_ = 0.06790 (p = 0.795)	0.0003	0.058
**9(S)-HODE [μg/ml]**	X = 0.107 SD = 0.120	X = 0.107 SD = 0.069	X = 0.086 SD = 0.049	X = 0.075 SD = 0.044	**intercept**	**F_1216_ = 250.7985 (p < 0.001)**	**0.537**	**1.000**
					**BPH/control**	**F_1216_ = 5.0326 (p = 0.026)**	**0.023**	**0.608**
					**MetS**	F_1216_ = 0.2255 (p = 0.635)	0.003	0.076
					BPH/control*MetS	F_1216_ = 0.2496 (p = 0.618)	0.001	0.079
**5-oxo-ETE [μg/ml]**	X = 0.579 SD = 1.233	X = 0.431 SD = 0.453	X = 0.233 SD = 0.213	X = 0.255 SD = 0.235	**intercept**	**F_1216_ = 46.63848 (p < 0.001)**	**0.178**	**0.999**
					**BPH/control**	**F_1216_ = 5.47664 (p < 0.001)**	**0.025**	**0.644**
					**MetS**	F_1216_ = 0.36091 (p = 0.549)	0.002	0.092
					BPH/control*MetS	F_1216_ = 0.56305 (p = 0.454)	0.003	0.116
**5-HETE [μg/ml]**	X = 0.234 SD = 0.531	X = 0.431 SD = 0.453	X = 0.089 SD = 0.026	X = 0.096 SD = 0.030	**intercept**	**F_1216_ = 162.4929 (p < 0.001)**	**0.429**	**1.000**
					**BPH/control**	**F_1216_ = 8.6798 (p = 0.004)**	**0.039**	**0.835**
					**MetS**	F_1216_ = 0.4566 (p = 0.500)	0.002	0.103
					BPH/control*MetS	F_1216_ = 0.0905	0.0004	0.060

## DISCUSSION

The state of knowledge of BPH and its pathophysiology is improving. This topic is still subject to research and numerous discussions. In our investigation, we analyze as many lipid-derived inflammatory markers in men with BPH. The coexistence of MetS in these patients was also taken into account in order to find out whether the examined parameters can be involved in the pathophysiology of BPH and MetS.

The primary risk factors for the development of BPH are hormonal disorders [39, 40], MetS, obesity, and other metabolic disorders, as well as chronic inflammation [41]. Inflammation is an orderly, physiological process resulting from the immune system, i.e. the type of defense of the body in response to a damaging factor and the subsequent healing of tissues [42]. Chronic inflammation can lead to tissue damage, pathology, and disease. Changes in the blood vessels are the underlying cause of the inflammatory reaction (when vasodilation occurs, and tissue blood supply and vascular permeability increase, leukocytes or plasma proteins can enter the inflamed area). Chemical mediators are then released in the region of the inflammatory reaction. Many types of inflammatory cells and mediators are involved in this process [43]. Depending on the nature of the inflammatory stimulus, the anatomical site, and the type of inflammatory response, these mediators can include lipids, peptides, or amino acid derivatives.

As essential nutrients, fatty acids are involved in metabolism and cellular biochemical transformations. PUFAs are important components of the phospholipids of all cells. Improper regulation of fatty acids can cause inflammation and carcinogenesis [44]. PUFAs regulate inflammatory processes through various mechanisms, which include participating as mediators or altering the composition of cell membranes. The primary mediators of inflammation are PUFA derivatives specifically arachidonic acid (AA) and linoleic acid (LA) [45, 46], as well as their oxidation products including prostaglandin (PG), thromboxane (TX), 5-oxo-eicosatetraenoic acid (5-oxo-ETE), 12- and 15-hydroxyeicosatetraenoic acid (HETE), and 9- and 13-hydroxyoctadecadienoic acids (HODEs) [45, 46]. PUFAs are found in frequently eaten foods, and their overconsumption can lead to higher levels of prostaglandins in the body [46]. The content of AA and n-3 fatty acids―eicosapentaenoic acid (EPA) and docosahexaenoic acid (DHA)―can be changed by their exogenous administration [46]. Fatty acids, activated by acyl-CoA synthetase, can then undergo beta oxidation to acetyl-COA in the mitochondrion or be attached to triglycerides or phospholipids [44, 47]. The metabolism of AA depends, among other things, on acyl-CoA synthetase long-chain family member 9. In addition, higher concentrations of AA contribute to a decrease in the activity of cyclo-oxygenase-2 (COX-2), but stimulate the processes in which lipoxygenase (LOX) is involved. This, in turn, reduces the concentration and activity of prostaglandin E2, and stimulates the production of lipoxin A4 (LXA_4_).

Our research showed that men with BPH had lower prostaglandin E2 levels than healthy controls. Similarly, Fowke et al. (2016) [48] did not confirm the relationship between prostate hyperplasia and increased prostaglandin E2 levels. Some authors have confirmed the involvement of prostaglandin E2 in the process of prostate cancer formation and its metastasis to other organs [49, 50]. Nevertheless, the role of prostaglandin E2 seems to be still unclear, as Kiely et al. (2021) [51] do not confirm the relationship between higher prostaglandin E2 levels and prostate cancer progression.

LA is the main component of LDLs. Elevated LDL levels combined with oxidative stress and antioxidant deficiency stimulate oxidative processes, playing an important role in the regulation of inflammatory processes. LA also affects the processes within respiratory tract smooth muscles and the vascular wall, thereby regulating pain sensitivity and endogenous steroid hormones associated with the etiopathogenesis of metabolic syndrome disorders. The metabolic effects of LA derivatives, which include HODEs, oxo-octadecadienoic acids (oxo-ODE), epoxy-octadecadienoic acid, and epoxy-keto-octadecenoic acids, remain unexplained.

They have a pleiotropic effect (beneficial or deleterious), and so it is impossible to identify a clear answer. As mentioned earlier, prostaglandin and thromboxane are produced from AA (omega-6 (ω-6) polyunsaturated fatty acid), which may suggest that eating foods high in ω-6 fatty acids may induce or inhibit inflammatory processes [52].

HETEs and HODEs are involved in the inhibition of granulocyte aggregation, and dilate blood vessels, thereby affecting inflammatory responses in the pathogenesis of atherosclerosis and MetS [24, 46].

This, in turn, may be related to urological diseases (including BPH), which are known to be associated with inflammation [53]. It is believed that dietary fatty acids and their derivatives, by modulating inflammatory reactions, may affect the remodeling of the prostate epithelium, and consequently the functioning of the gland, thus increasing susceptibility to its disorders [54].

Our study of lipid markers of inflammation revealed statistically significant differences in 5-oxo-ETE levels between the male groups. 5-Oxo-ETE is one of the more potent ‘agents’ for cell signaling, involved in autocrine and paracrine regulation. It has also been postulated that 5-oxo-ETE contributes to the local inflammatory response and stimulates cancer cell proliferation. It may also play a role as a pro-inflammatory mediator in diabetes [24].

Men with BPH exhibited statistically significantly higher concentrations of 12-HETE. It had previously been reported that 12-HETE levels were higher in the urine of BPH patients than of men without prostate disease [55, 56]. Moreover, Dilly et al. (2013) [57] demonstrated that in prostate tumor cells, 12-HETE stimulates the release of matrix metalloproteinase 9 (MMP-9), which plays a significant role in the process of tumor angiogenesis. There is also a link between the development of prostate adenocarcinoma and 12-HETE. This may indicate that this marker is involved in the formation of benign and malignant lesions in the prostate. This marker may also inhibit platelet aggregation induced by thromboxanes A2 (TXA2), leading to platelet destruction mediated by reactive oxygen species (ROS) [58]. Furthermore, it may serve as a mediator for some of the pro-inflammatory and pro-apoptotic effects of specific cytokines involved in the pathogenesis of diabetes [59], which is a component of MetS. However, we were unable to show statistically significant differences in the concentrations of this marker depending on MetS.

Our study also demonstrated statistically significant higher levels of 15-HETE in patients with BPH. To date, evidence has emerged to suggest that 15-HETE may be involved in the regulation of vascular smooth muscle and endothelial cell function, cell proliferation, and the development of inflammation [24, 60]. There is also evidence for the involvement of this marker in the neoplastic process in the prostate [61].

We showed that serum 9-HODE concentrations were statistically significantly higher in men with BPH compared to controls. According to Spiteller et al. (1998) [62], this marker is associated with oxidative stress, inflammation, and numerous pathological conditions. Studies show links to atherosclerosis, diabetes, Alzheimer’s disease, NAFLD, psoriasis, chronic inflammation, obesity, and cancer. To date, no studies have been conducted in patients with BPH.

In our study, we also presented scientific evidence for the involvement of the following markers in BPH: 5(S),6(R),15(R)-LTXA4 and 5-HETE, whose serum concentrations in men with BPH were statistically significantly higher than in those without BPH. Moreover, the co-existence MetS was proven to affect the concentrations of 5(S), 6(R)-LTXA4 in the study group. Due to the lack of scientific evidence in the literature, the role of these markers in the pathogenesis of BPH remains unclear.

A breakthrough in understanding the pathophysiology of inflammation was made with the discovery of special lipid mediators associated with endogenous alleviation of inflammation. This is an active process involving endogenous anti-inflammatory and proresolving lipid mediators [63]. They include several groups of mediators known as lipoxin (LX), resolvin (Rv), protectin (PD), and maresin (MaR). Lipoxins are derived from ω-6 acids, and resolvins, protectins, and maresins from ω-3 acids [64].

The findings of our study indicated the existence of statistically significant discrepancies in the concentrations of resolvin E1, protectins D1, and maresins 1. Patients with BPH exhibited elevated serum concentrations of the aforementioned biomarkers. Studies suggest that lipid mediators are involved in alleviating inflammatory and allergic reactions, wound healing, and relieving neuropathic pain. So far, a protective effect of resolvin E1 against bronchial asthma and complications in the form of respiratory tract infections has been described [65]. Based on a mouse model study, resolvins E1 exhibit anti-inflammatory and analgesic effects, by inhibiting the expression of pro-inflammatory cytokines, neutrophil infiltration, and TNF-α activity [66]. Maresin 1 increases the uptake of apoptotic neutrophils by macrophages, stimulates macrophage phagocytosis, and reduces neutrophil penetration into inflammatory foci. It also inhibits the production of leukotriene B4―an AA metabolite―by direct inactivation of the enzyme, leukotriene A4 hydrolase (LTA4H). The effect may be suppression of the inflammatory response by reducing the production of the pro-inflammatory mediator, leukotriene B4 [67].

## CONCLUSIONS

Despite new information and evidence on specialized lipid mediators, the existing literature remains deficient in its analysis of mediators in diverse models of inflammation, and their potential contribution to the pathophysiology of chronic diseases. BPH is the result of many factors that reinforce each other’s adverse effects on the prostate gland. Endocrine disorders cannot be considered as the only factors determining the development of BPH [68]. It is evident that androgens exert a pivotal influence on the formation of the male genitourinary tract, facilitating the differentiation and proliferation of stromal and prostatic epithelial cells. The findings of the present study indicate that pro-inflammatory mediators and suppressors of inflammation may play a role in the in the development of BPH, but their exact contribution has yet to be investigated.

## MATERIALS AND METHODS

### Patients

The study was conducted with the participation of 144 men diagnosed and treated for BPH who underwent transurethral resection of the prostate (TURP) at the Department of Urology and Urological Oncology, Pomeranian Medical University, Szczecin, Poland. The mean age of the men was 66.326 ± 6.479 years. The diagnosis was based on the following criteria: increased prostate volume (>30 ml), decreased urinary flow (Q_max_) or urinary retention, International Prostate Symptom Score (IPSS) results, and long-term symptoms. Furthermore, the presence of benign prostatic hyperplasia was confirmed in the prostate tissue excised during the TURP procedure.

The control group consisted of healthy volunteers (n = 75) at the age of 54.66 ± 6.523 years with prostate size ≤ 30 ml and PSA < 2.5 ng/ml. The patients in this group did not report any symptoms of BPH, and their IPSS scores were less than or equal to seven points.

Patients were excluded from participation in the study if they had any of the following conditions: active cancer, alcoholism, thyroid disease, or had been taking glucocorticosteroids for a period of six months prior to the study. Only those patients from whom serum was collected for testing were included in the study.

### Clinical examination

Anthropometric measurements including body weight, height, and waist circumference were conducted on all patients enrolled in the study. Participants were also asked to provide information regarding their demographic characteristics and health status. The male participants were divided into groups based on the presence of metabolic syndrome, which was diagnosed in accordance with the 2005 International Diabetes Federation (IDF) criteria.

### Blood serum analysis

Blood samples were collected from patients for the purpose of determining basic biochemical parameters, including serum glucose, total cholesterol, LDL cholesterol, HDL cholesterol, and triglycerides. Blood samples (7.5 ml) were collected from subjects in a fasting state between 7:30 and 9:00 a.m. from the cubital vein. The blood was collected by qualified medical personnel, and the material for testing was delivered to the laboratory in accordance with appropriate rules and procedures. The serum was subsequently collected and stored at −80° C the lipid marker analysis could be conducted. The biochemical parameters in serum were determined spectrophotometrically using commercially available reagent kits.

### Eicosanoid determination procedures

### Eicosanoid isolation

The analysis of fatty acid derivatives was performed on the serum of patients. To this end, 0.5 ml of plasma was collected into test tubes, to which 1 ml of 100% acetonitrile and 50 μl of an internal standard, prostaglandin B2 (PGB2, Sigma-Aldrich, Merck KGaA, Darmstadt, Germany), were added. Subsequently, the samples were isolated at a temperature of -20° C for a period of 15 minutes. Following the designated period, the samples were centrifuged at 10,000 rpm for 10 minutes at 4° C (Eppendorf, Centrifuge 5804R). The resulting pellet was discarded, and the supernatant was transferred to new tubes and mixed with 4.5 ml of 1 mM HCl. In order to attain a pH of 3, 30–50 μl of 1 M HCl was also added to the tubes.

During the incubation period, RP-18 C18 solid-phase extraction (SPE) columns (Agilent Technologies, USA) were prepared for isolation. The columns were activated by rinsing first with 3 ml of 100% acetonitrile and then with 3 ml of 20% acetonitrile in 1 mM hydrochloric acid. The samples were then applied to the columns and subsequently washed with 3 ml of a 20% acetonitrile solution in 1 mM hydrochloric acid. Fatty acid derivatives were eluted from the columns using 1.5 ml of a methanol acetate solution (1:1, v:v). The samples were then dried under vacuum and dissolved in 100 μl of 0.01% acetic acid (Sigma-Aldrich, Merck KGaA, Germany) in 60% methanol. The eicosanoids were separated using liquid chromatography.

### High-performance liquid chromatography (HPLC) analysis

The separation was performed on an Agilent Technologies 1260 liquid chromatograph (Santa Clara, CA, USA) equipped with a Thermo Scientific Hypersil BDS C18 column (100 × 4.6 mm, 2.4 μm) and a UV-vis detector. The following fatty acid derivatives were identified: LXA_4_ 5S,6R (cat no. 20110), LXA_4_ 5S,6R,15R (cat no. 20110), 5-HETE (cat no. 34230), 5-oxo-ETE (cat no. 34250), 12-HETE (cat no. 34570), 15-HETE (cat no. 34720), 16R/S-HETE (cat no. 10004385/10004384), 9-HODE (cat no. 38410), 13-HODE (cat no. 38610), 18-HEPE (cat no. 3284), 10S,17R-DiDHA (Protectin D1) (cat no. 10008128), MaR1 (cat no. 10878), LTB4 (cat no. 20110), PGB2 (cat no. 11210), PGE2 (cat no. 14010), RvD1 (cat no. 10012554), and RvE1 (cat no. 10007848).

A flow rate of 1 ml/min and an injection volume of 60 μl were used. The separation was conducted using a gradient method with solvent A (methanol:water acid, 50:50:0.1, v:v) and solvent B (methanol:water acid, 100:0:0.1, v:v). Initially, the gradient started with 30% solvent B for 2 minutes, followed by 80% from 2 to 33 minutes, 98% from 33.1 to 37.5 minutes, and 30% from 40.3 to 45 minutes. The compounds were analyzed on ChemStation software (Agilent Technologies, Cheadle, UK) using a calibrated method.

### Statistical analysis

The results of the study were statistically analyzed using Statistica v. 13.3 (TIBCO Software Inc.). Mean, median (Me), minimum (Min) and maximum (Max) values, and standard deviation (SD) were calculated for all measurable variables in both groups. The normality of the distribution of the variables was then tested using the Shapiro-Wilk test. To compare the independent variables of both groups, Student’s t test for variables with normal distribution and the Mann-Whitney U test for variables without normal distribution were used. Additionally, multivariate ANOVA was used. Pearson’s correlation analysis was used to determine the relationship between the variables - the correlation was regarded as statistically significant when the correlation coefficient (r) was different from zero and when the level of statistical significance was p ≤ 0.05. Pearson’s chi-square test was used to compare nominal data. The level of statistical significance was set at p ≤ 0.05.

### Limitations of the study

One limitation of the study is the age difference between the study group and the control group. Nevertheless, both groups consist of aging men. The results we obtained motivate us to conduct further analyses with the inclusion of new variables, to make the research more precise.
